# Summary of World Café Discussions Table 2: Evaluation

**DOI:** 10.25646/6508

**Published:** 2020-06-04

**Authors:** Sebastian Haller, Henriette Steppuhn

**Affiliations:** 1 Robert Koch Institute, Berlin, Department of Infectious Disease Epidemiology; 2 Robert Koch Institute, Berlin, Department of Epidemiology and Health Monitoring

Evaluation may be conducted prior to or after implementation of public health interventions. The objectives of both evaluation types differ accordingly. While pre-implementation evaluation aims at gathering available information in order to decide which interventions should be recommended or implemented, post-hoc evaluation focusses on determining benefits and harms of existing public health interventions. Considering these introductory remarks, we worked on the question: ‘Which methods, skills, and data does the Robert Koch Institute (RKI) as a national public health institute need to perform evaluations of public health interventions?’ The main discussion centered on strengths and weaknesses of different methods according to type of evaluation and potential application opportunities.

Firstly, ex-ante evaluation has already become an integral part in the work of several working groups at the RKI. Systematic reviews, risk of bias assessment, appraisal of reliability and validity of evidence are regularly conducted and tools for informed decision making are used for STIKO (German Standing Committee on Vaccination) recommendations. In future, methodological advancements in performing evidence synthesis such as overviews of reviews or other forms of abbreviated literature reviews might facilitate well-timed evidence-based policy advising and information of the public.

Secondly, post-hoc evaluation e.g. of recommendations on vaccinations or health targets (Gesundheitsziele) also constitute core tasks of the RKI. Appropriate methods may be chosen from the broad spectrum of epidemiological methods. Among these, interrupted time series studies have, so far, been conducted e.g. in the outcome evaluation of recommendations on rotavirus vaccination. In future, use of other high-quality quasi-randomized study designs e.g. instrumental variable or regression discontinuity design might provide further insight into the causal impacts of interventions. Moreover, modelling of benefits and harms of interventions can be used for projections into the future. This allows for capturing the full value of an intervention not only on health but also on non-health outcomes.

Finally, to implement a public health intervention in a new setting may be challenging. Also, benefits and harms of interventions may differ substantially according to context. Lacks of external validity of existing data therefore need to be adequately addressed. Qualitative studies may be useful to understand which intervention works for whom and why. Further qualitative methods can be applied to illustrate particularities of another setting and find appropriate modifications for the interventions. The skills needed for such process evaluation research are even more diverse than for mere effectiveness studies. Thus interdisciplinary cooperation is of great value. Evidence public health networks may allow exchange of experiences and methodological skills. We should avoid redundant processes and improve systematic gain of knowledge.

